# Administration of cardiac mesenchymal cells modulates innate immunity in the acute phase of myocardial infarction in mice

**DOI:** 10.1038/s41598-020-71580-z

**Published:** 2020-09-08

**Authors:** Yi Kang, Marjan Nasr, Yiru Guo, Shizuka Uchida, Tyler Weirick, Hong Li, Jae Kim, Joseph B. Moore, Senthilkumar Muthusamy, Roberto Bolli, Marcin Wysoczynski

**Affiliations:** 1 Diabetes and Obesity Center, University of Louisville School of Medicine, 580 South Preston St. – Rm 204B, Louisville, KY 40202 USA; 2 Institute of Molecular Cardiology, University of Louisville School of Medicine, Louisville, KY USA; 3 Cardiovascular Innovation Institute, Univerity of Louisville School of Medicine, Louisville, KY USA

**Keywords:** Immunology, Inflammation, Innate immune cells, Innate immunity

## Abstract

Although cardiac mesenchymal cell (CMC) therapy mitigates post-infarct cardiac dysfunction, the underlying mechanisms remain unidentified. It is acknowledged that donor cells are neither appreciably retained nor meaningfully contribute to tissue regeneration—suggesting a paracrine-mediated mechanism of action. As the immune system is inextricably linked to wound healing/remodeling in the ischemically injured heart, the reparative actions of CMCs may be attributed to their immunoregulatory properties. The current study evaluated the consequences of CMC administration on post myocardial infarction (MI) immune responses in vivo and paracrine-mediated immune cell function in vitro. CMC administration preferentially elicited the recruitment of cell types associated with innate immunity (e.g., monocytes/macrophages and neutrophils). CMC paracrine signaling assays revealed enhancement in innate immune cell chemoattraction, survival, and phagocytosis, and diminished pro-inflammatory immune cell activation; data that identifies and catalogues fundamental immunomodulatory properties of CMCs, which have broad implications regarding the mechanism of action of CMCs in cardiac repair.

## Introduction

Although cell therapy is a promising and safe approach for the treatment of heart failure (HF)^1,2^ , the mechanism whereby injected cells improve heart function remains poorly understood. Preclinical studies have shown that improvement of left ventricular (LV) function after cell therapy is associated with increased angiogenesis and reduction of fibrosis and inflammation^1,3–7^. It is now well accepted that injected cells do not improve heart function by differentiating into cardiomyocytes and endothelium^1,8–12^. The negligible long-term survival of injected cells suggests that cell therapy activates endogenous mechanisms of repair via secretion of paracrine factors^1,6–12^, the nature of which, however, remains unclear.

Myocardial damage due to ischemic injury elicits an inflammatory response^13–15^. In the first phase, neutrophils and pro-inflammatory monocytes are recruited to facilitate the degradation of dead tissue via secretion of proteolytic enzymes^16–19^. In the subsequent few days, the neutrophil response resolves and monocytes differentiate to reparative macrophages which remove degraded dead tissue and apoptotic cells via efferocytosis, but also secrete cytokines to facilitate angiogenesis and activate fibroblasts^13,14^. Activation of fibroblasts and their conversion to myofibroblasts facilitates the deposition of extracellular matrix and scar formation and also results in resolution of inflammation^13–15,20–22^. A dysregulated immune response leads to the failure of timely scar formation which results in expansion of the scar area and further damage of the remaining cardiac muscle. Moreover, systemic depletion of neutrophils or macrophages leads to disarrangement of the reparative immune response, failure of infarct healing, and scar expansion leading to cardiac rupture^18,19,23–25^. Thus, immune cells are indispensable for the wound healing response to ischemic injury. Because cell therapy has salutary effects on cardiac recovery after myocardial infarction (MI), it can be hypothesized that injected cells activate the reparative immune response to preserve cardiac function.

We have previously demonstrated that cardiac mesenchymal cells (CMCs) exert significant reparative actions after acute MI^5,26^. Although the immune system is a necessary component of myocardial repair, it is unknown whether the improvement in cardiac function after cell therapy is due to immunoregulatory actions of CMCs. Therefore, the purpose of this study was to determine whether administration of CMCs soon after MI regulates the reparative immune response. We found that CMC injection recruits innate immune cells (monocytes, macrophages, neutrophils) but has no effect on adaptive immunity (B and T cells). In vitro data demonstrated that the secretome of CMCs exerts chemotactic activity towards neutrophils and monocytes/macrophages, enhances survival of neutrophils and macrophages, enhances phagocytosis, and inhibits pro-inflammatory activation of macrophages. These observations suggest that CMCs have immunomodulatory properties that could contribute to CMC-induced myocardial repair. Identification of reparative immune pathways enhanced by cell therapy would advance our understanding of the mechanism of action of cell therapy and may provide a new therapeutic target for improving myocardial repair without administration of cell products.

## Materials and methods

### Isolation and culture of CMCs

All procedures were approved by the University of Louisville Institutional Animal Care and Use Committee and were in accordance with NIH guidelines. The euthanasia procedures were consistent with the AVMA Guidelines for the Euthanasia. CMCs were isolated as previously described^5^. Briefly, myocardial cells were isolated from 12 to 15 weeks old, male C57BL6 mice (The Jackson Laboratory). Mice were euthanized by sodium pentobarbital injection (100 mg/kg i.p.). The hearts were excised, washed in room temperature PBS, minced into small pieces, and enzymatically dissociated with Collagenase II (5 mg/mL in PBS; Worthington) with gentle agitation for 45 min at 37 °C. After Collagenase II inactivation with DMEM/F12 medium containing 10% FBS, cells were centrifuged at 600×*g* for 10 min. The collected cell pellet was suspended in growth medium consisting of DMEM/F12 (Invitrogen), 10% FBS (Seradigm, VWR), bFGF (10 ng/ml), EGF (10 ng/ml), ITS (insulin/transferrin/selenium), glutamine, and Pen-Strep. The CMCs were cultured and passaged twice before cryopreservation in liquid nitrogen. Upon thawing, the cells were propagated in growth media for 2–6 passages prior to in vitro or in vivo experiments.

### Murine model of MI

Studies were performed in C57BL6/J female mice (age 12–15 weeks) purchased from The Jackson Laboratory (Bar Harbor, ME, USA). Mice were maintained in microisolator cages under specific pathogen-free conditions in a room with a temperature of 24 °C, 55–65% relative humidity, and a 12-h light–dark cycle. The murine model of myocardial ischemia and reperfusion has been described in detail^5,26^. Briefly, mice were anesthetized with sodium pentobarbital (60 mg/kg i.p.) and ventilated using carefully selected parameters. The chest was opened through a midline sternotomy, and a nontraumatic balloon occluder was implanted around the mid-left anterior descending coronary artery using an 8–0 nylon suture. To prevent hypotension, blood from a donor mouse was given at serial times during surgery. Rectal temperature was carefully monitored and maintained between 36.7 and 37.3 °C throughout the experiment. In all groups, MI was produced by a 60-min coronary occlusion followed by reperfusion. Successful performance of coronary occlusion and reperfusion was verified by visual inspection (i.e., by noting the development of a pale color in the distal myocardium after inflation of the balloon and the return of a bright red color due to hyperemia after deflation) and by observing S-T segment elevation and widening of the QRS on the ECG during ischemia and their resolution after reperfusion).

### Preparation of cells for injection

As previously described^4^, on the day of injection, cells were 60–80% confluent. The cell monolayer was washed two times with room temperature PBS to remove debris and dead cells. Subsequently, cells were harvested by enzymatic digestion with 0.25% trypsin–EDTA (Invitrogen). The resulting cell suspension was trypsin-inactivated with DMEM/F12 supplemented with 10% FBS. After centrifugation at 600×*g* for 10 min at RT, cells were suspended in PBS and counted using a hemocytometer. Next, cells were filtered through a 40-μm filter and centrifuged for 10 min at 600×*g* at RT. A total of 0.5 × 10^6^ cells were suspended in 40 µL of sterile PBS (pH 7.4) at room temperature and transported to the mouse surgery laboratory for injection. Extra cell suspension volume was prepared to account for the dead space in the syringe and needle used for injections.

### Intramyocardial cell delivery

At 48 h after MI, mice were anesthetized and the chest reopened through a central thoracotomy. Cells (0.5 × 10^6^ cells in 40 μL) or an equivalent volume of PBS vehicle were injected intramyocardially using a 30-gauge needle. A total of four injections (10 μL each) were made in the peri-infarct region in a circular pattern, at the border between infarcted and noninfarcted myocardium, as described^5^.

### Flow cytometric analysis in vivo

At 2 d after MI or sham operation, or at 7 d after cell or vehicle injection (9 d after MI), mice were euthanized by sodium pentobarbital injection (100 mg/kg i.p.). The hearts were excised and perfused with cold PBS for 10 min to wash out peripheral blood, minced into small pieces, enzymatically dissociated with Collagenase II (5 mg/mL in PBS; Worthington) with gentle agitation for 45 min at 37 °C, and washed with PBS. Next, myocardial cells were isolated at the interface of 70% and 30% of discontinuous Percoll gradient after centrifugation at 1,750×*g* at room temperature for 20 min. Subsequently, collected cells were washed in PBS and stained with a cocktail of antibodies. The following monoclonal antibodies were used for flow cytometry analyses: anti-Ly-6G (eBioscience, clone 1A8), anti-CD11b (eBioscience, clone M1/70), anti-CD115 (eBioscience, clone AFS98), anti-Ly6C (eBioscience, clone HK1.4), anti-CD3 (eBioscience, clone 145-2C11), anti-CD8a (eBioscience, clone 53–6.7), anti-CD4 (eBioscience, clone GK1.5), anti-CD19 (eBioscience, clone 1D3), anti-CD90.2 (eBioscience, clone 30H12), anti-NK1.1 (eBioscience, clone PK136), anti-CD49b (eBioscience, cloneDX5), anti-MHC Class II (eBioscience, clone AF6-120.1), anti-CD11c (eBioscience, clone N418). Cells were identified as: two populations of macrophages (reparative macrophages: Lin^low^CD11b^pos^F4/80^pos^Ly6C^low^; pro-inflammatory macrophages: Lin^low^CD11b^pos^F4/80^pos^Ly6C^high^), two populations of monocytes (reparative monocytes: Lin^low^CD11b^pos^F4/80^neg^MHCII^neg^CD11c^neg^Ly6C^low^; pro-inflammatory monocytes: Lin^low^CD11b^pos^F4/80^neg^MHCII^neg^CD11c^neg^Ly6C^high^), and two populations of neutrophils (pro-inflammatory N1 neutrophils: CD11b^pos^Ly6G^pos^CD206^neg^; reparative N2 neutrophils: CD11b^pos^Ly6G^pos^CD206^pos^). The data analysis was performed with FlowJo software.

### Isolation of bone marrow macrophages

12–15-weeks old, male C57BL6 mice (The Jackson Laboratory) were euthanized by sodium pentobarbital injection (100 mg/kg i.p.). Tibia and femur bones were flushed with ice-cold PBS using a 21-gauge needle. The collected bone marrow cell pellet was suspended in a growth medium consisting of DMEM/F12 (Invitrogen), 10% FBS (Seradigm, VWR), M-CSF (50 ng/ml), and Pen-Strep. A single-cell suspension was plated in the tissue culture flask. After 24 h, floating cells were removed and adherent cells were expanded for 7–10 d with media change every other day. Macrophage purity was determined by co-expression of CD11b and F4/80 markers, evaluated with flow cytometry.

### Isolation of bone marrow neutrophils

Bone marrow cells were isolated from tibia and femur bones as described above (isolation of bone marrow macrophages). Bone marrow neutrophils were isolated with a Neutrophils isolation kit (Miltenyi Biotech), according to manufacturer’s recommendations. Briefly, neutrophils were isolated by depletion of non-target cells. Non-target cells were labeled with a cocktail of biotin-conjugated monoclonal antibodies, as primary labeling reagent, followed by anti-biotin monoclonal antibodies conjugated to MicroBeads as a secondary labeling reagent. The magnetically labeled non-target cells were depleted by retaining them on LS MACS Column in the magnetic field of a MACS separator, while unlabeled neutrophils ran through the column. The neutrophils' purity was evaluated by CD11b and Ly6G co-expression with flow cytometry, as described^27^.

### Real-time PCR

As described^28^, total mRNA was isolated with the RNeasy Mini Kit (Qiagen) and reverse-transcribed with TaqMan Reverse Transcription Reagents (Applied Biosystems). Quantitative assessment of mRNA pro-inflammatory (*IL1b*, *Il6*, *Tnfa*, *Ccl2*, *Ccl5*) and anti-inflammatory markers (*Il10*, *Tgfb*,*1*, *Thgf2*, *Tgfb3)* and β2-microglobulin was performed by qRT-PCR using StepOne (Applied Biosystems). A 10-μL-reaction mixture containing 5 μL of SYBR Green PCR Master Mix and forward and reverse primers was used. The threshold cycle (Ct), i.e., the cycle number at which the amount of amplified gene of interest reached a fixed threshold, was subsequently determined. Relative quantitation of mRNA expression was performed with the comparative Ct method. The relative quantitative value of the target, normalized to an endogenous control (β2-microglobulin gene) and relative to a calibrator, was expressed as 2^−ΔΔCt^ (-fold difference), where ΔCt = (Ct of target genes]) − (Ct of endogenous control gene [β2-microglobulin]), and ΔΔCt = (ΔCt of samples for target gene) − (ΔCt of calibrator for the target gene). To avoid the possibility of amplifying contaminating DNA (1) all of the primers for real-time RT-PCR were designed to contain an intron sequence for specific cDNA amplification; (2) reactions were performed with appropriate negative controls (template-free controls); (3) uniform amplification of the products was rechecked by analyzing the melting curves of the amplified products (dissociation graphs); and (4) the melting temperature (Tm) was 57–60 °C, and the probe Tm was at least 10 °C higher than primer Tm.

### Statistical analysis

Results are shown as mean ± SEM. Statistical analyses (GraphPad 7.0d) were performed with Student’s t-tests or one-way ANOVA followed by Student’s *t* tests with Bonferroni correction, as appropriate. Differences were considered statistically significant when *P* < 0.05.

## Results

The magnitude and kinetics of immune cell infiltration are highly dependent on the model of myocardial injury. Compared with non-reperfused MI models, ischemia followed by reperfusion is associated with a reduction of infiltrating leukocytes and tends to shift the kinetics of the immune response towards faster resolution^17^. Therefore, first, we profiled the immune cells in the myocardium at the time of CMC injection in our model of 60-min ischemia followed by reperfusion. Single-cell suspensions of digested hearts at 2 d after MI or sham operation were labeled with fluorescently labeled antibodies.

### Profile of myeloid immune cells 2 d after MI

**Figure 1:**
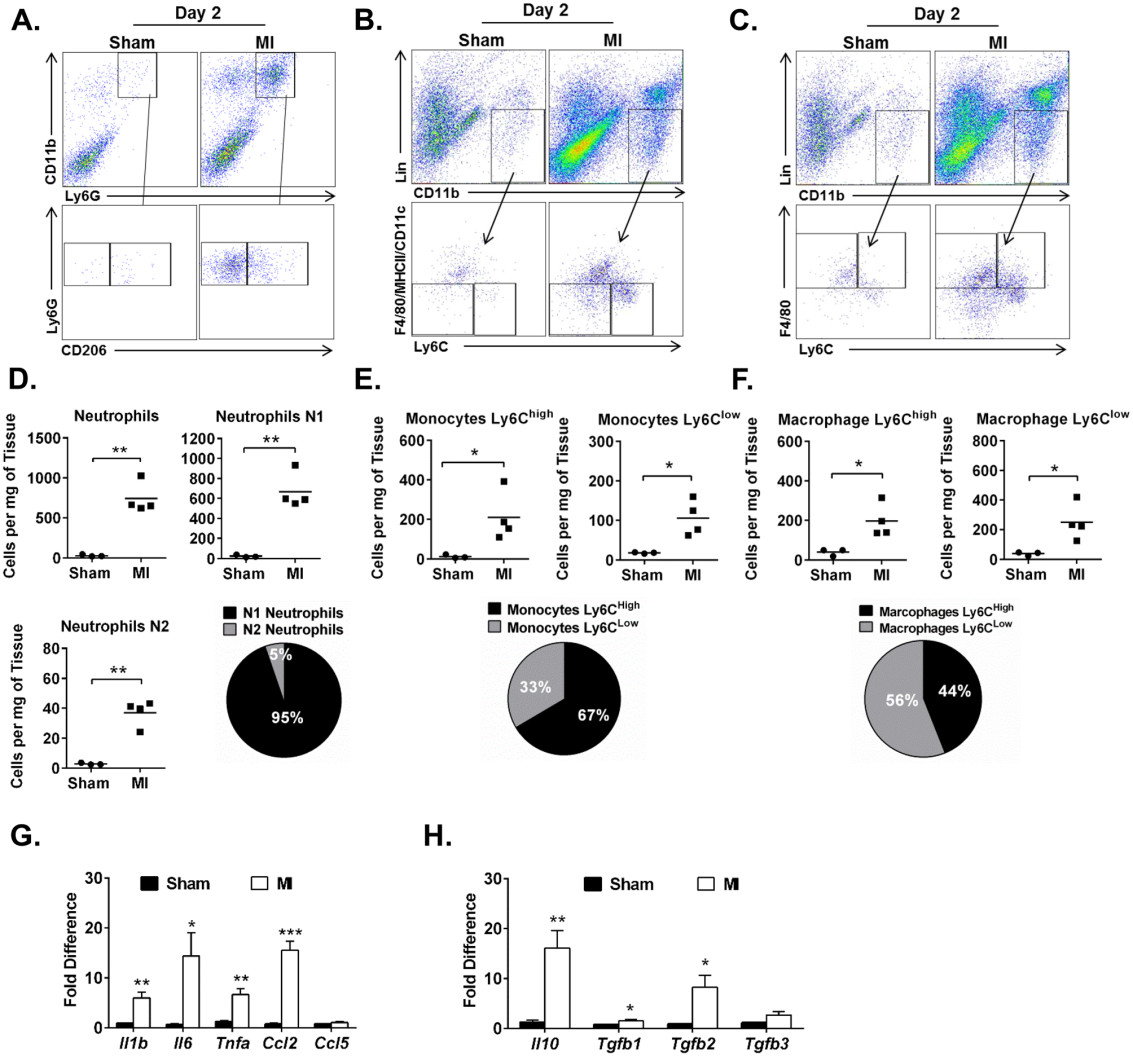
Mouse hearts 2 d after MI are infiltrated in pro-inflammatory immune cells. Cell suspensions of enzymatically digested hearts from sham and MI mice were stained with fluorescently labeled antibodies. Representative dot plots identified: (**A**) neutrophils as CD11b^pos^Ly6G^pos^ cells, which were stratified into N1 (CD206^neg^) and N2 (CD206^pos^) subpopulations; (**B**) monocytes Lin^neg^CD11b^pos^ (F4/80/CD11c/MHCII)^low^ with Ly6C^high^ and Ly6C^low^ expression; (**C**) macrophages Lin^neg^CD11b^pos^F4/80^high^ with Ly6C^high^ and Ly6C^low^ expression. Numerical representations of neutrophils (**D**), monocytes (**E**), and macrophages (**F**) and their subpopulations per mg of tissue; pie chart represents contribution of pro-inflammatory and anti-inflammatory cell subpopulations. Number of cells shown as mean; sham n = 3, MI n = 4; *P < 0.05; **P < 0.01 (*unpaired t test*). Quantitative PCR on mRNA isolated from hearts 2 d after sham and MI. Messenger RNA expression of pro-inflammatory (**G**) and anti-inflammatory (**H**) cytokines and chemokines in whole hearts 2 d after sham and MI. Data are means ± SEM; sham n = 4, MI = 4; *P < 0.05; **P < 0.01; ***P < 0.001 *(unpaired t test*).

Flow cytometry defined neutrophils as CD11b^high^Ly6G^high^ cells. These were further stratified into CD206^low^ and CD206^high^ subpopulations representing N1 and N2 neutrophils, respectively, (Fig. 1A), as previously described^29^. We found a low number of neutrophils in sham-operated mice (29.9 ± 7.7 cells per mg of tissue), but this number increased markedly in hearts after I/R injury (740.8 ± 96.2 cells per mg of tissue, *P* < 0.01). Characterization of neutrophil subpopulations showed a higher contribution of pro-inflammatory (N1) cells, which comprised 95% of total neutrophils in the heart after MI, compared with anti-inflammatory (N2) cells (667.9 ± 89.5 vs 37.1 ± 4.3 cells per mg of tissue, respectively, *P* < 0.01) (Fig. 1D). Next, flow cytometry detected monocytes as CD11b^high^ (CD90/B220/CD49b/NK1.1/Ly6G)^low^ (F4/80/MHCII/CD11c)^low^ cells, and divided them into two sub-populations: pro-inflammatory (Ly6C^high^) and anti-inflammatory (Ly6C^low^) monocytes (Fig. 1B), as previously described^18^. We found that compared with sham operation both Ly6C^high^ and Ly6C^low^ monocyte subpopulations were increased after MI (Ly6C^high^: 12.9 ± 5.5 vs 210.5 ± 62.7 cells per mg of tissue, respectively, *P* < 0.05; Ly6C^low^: 18.5 ± 1.0 vs 105.9 ± 22.6 cells per mg of tissue, *P* < 0.05). The pro-inflammatory subpopulation constituted ~ 67% of total monocytes present in the heart at 2 d after MI (Fig. 1E). Two subpopulations of macrophages with Ly6C^high^ and Ly6C^low^ expression were detected within the Lin^low^CD11b^high^F4/80^high^ population (Fig. 1C). Both subpopulations of macrophages were significantly increased in hearts after MI compared with sham-operated mice (Ly6C^high^, 40.1 ± 10.2 vs 196.4 ± 42.0 cells per mg, *P* < 0.05; and Ly6C^low^, 39.3 ± 7.3 vs 250.0 ± 61.7 cells per mg of tissue, *P* < 0.05) (Fig. 1F). These data indicate that at the time of CMC injection (2 d after MI), hearts are in the initial pro-inflammatory phase as indicated by high levels of neutrophils and pro-inflammatory monocytes.

### Profile of B and T lymphocytes 2 d after MI

Both T and B cells are involved in myocardial healing and regulation of immune response after MI^14,30–34^. Flow cytometry identified B cells in cell suspensions from hearts obtained 2 d after sham operation or MI as CD3^low^B220^high^ or CD3^low^CD19^high^. B cells, detected with both sets of markers, compared with sham-operated mice were elevated after MI (B220^high^, 5.4 ± 1.3 vs 35.7 ± 16.8 cells per mg of tissue; CD19^high^, 22.0 ± 2.9 vs 71.4 ± 23.2 cells per mg of tissue); however, these differences did not reach statistical significance (Supplementary Fig. 1). Next, we found a significant increase in total T CD3^pos^ cells in infarcted hearts (4.8 ± 0.6 vs 17.7 ± 4.1 cells per mg of tissue, *P* < 0.05). T cells were further stratified into cytotoxic (Tc) CD3^high^CD8^high^ and helper (Th) CD3^high^CD4^high^. Both populations were increased after MI, but only the Tc elevation reached statistical significance (Th, 0.5 ± 0.1 vs 2.6 ± 1.3 cells per mg of tissue; Tc, 1.1 ± 0.6 vs 7.9 ± 2.1 cells per mg of tissue, *P* < 0.05) (Supplementary Fig. 1). Previous reports have shown that B and T cell infiltration peaks at the time of resolution/repair and in the scar maturation phase^17^. The relatively low number of lymphocytes at 2 d after MI suggests that, at this time, hearts are still in the early, pro-inflammatory phase.

### Expression of pro- and anti-inflammatory factors 2 d after MI

To further examine the inflammatory milieu of the myocardium at the time of CMC injection, we evaluated the tissue expression of pro-inflammatory and anti-inflammatory cytokines with qPCR. We found augmented expression of the classical pro-inflammatory cytokines *Il1b*, *Il6*, *Tnfa* (~ 6.0, 14.4, and 6.7 fold increase, respectively) and the pro-inflammatory chemokine Ccl2 (~ 15.5-fold increase) in hearts 2 d after MI as compared with sham-operated controls (Fig. 1G). At the same time, expression of anti-inflammatory and pro-reparative cytokines (*Il10*, *Tgfb1*, *Tgfb2*, and *Tgfb3*) was increased (~ 16.0, 1.6, 8.2, and 2.7-fold increase, respectively) (Fig. 1H). The appearance of these anti-inflammatory cytokines at the mRNA level may indicate the beginning of the resolution of the inflammation phase after MI^14,20^.

### Intramyocardial injection of CMCs recruits neutrophils

**Figure 2:**
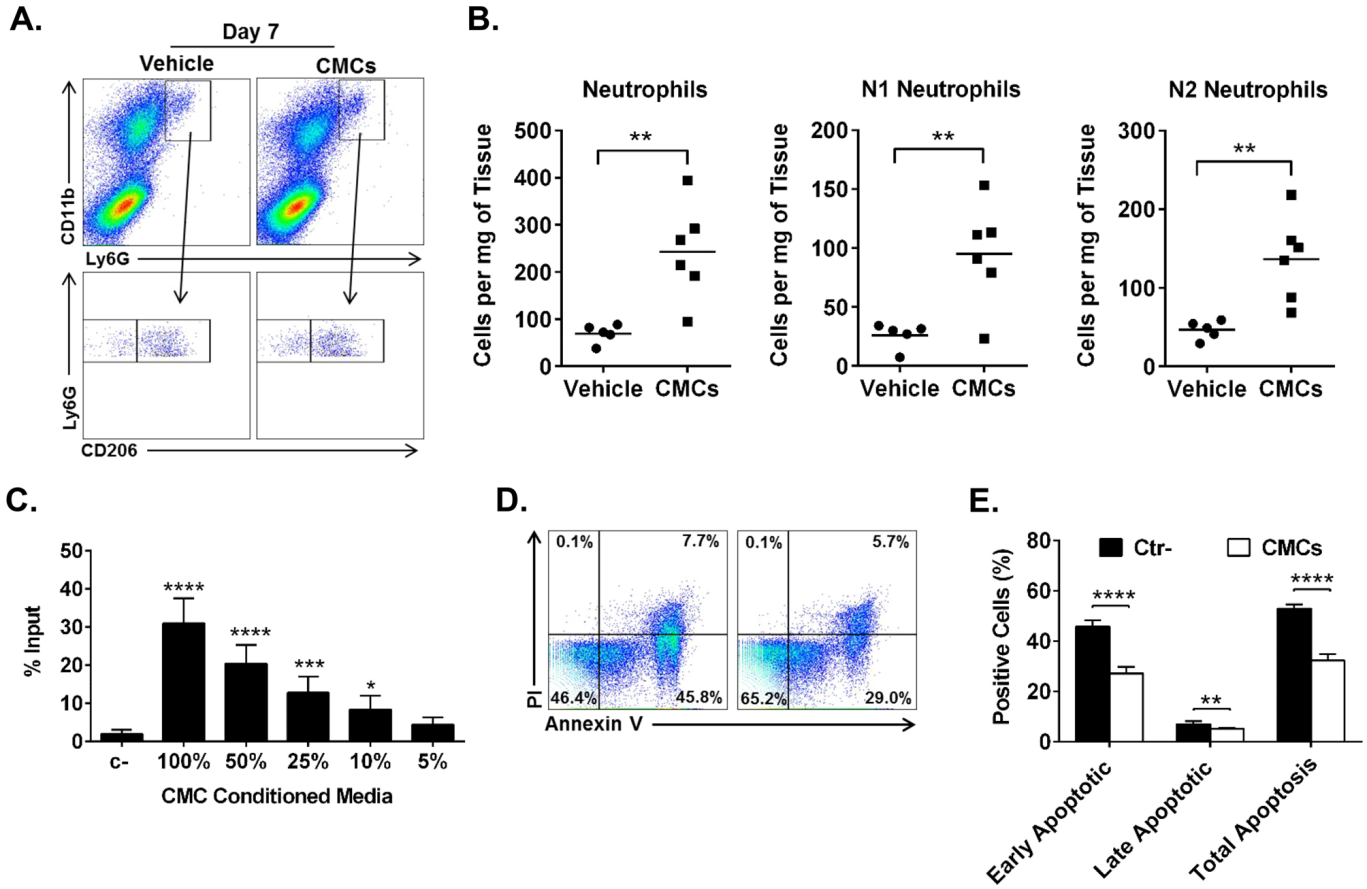
Effects of CMCs on neutrophils in vitro and in vivo. CMCs (5 × 10^5^) were injected in the mouse hearts 2 d after MI. Seven days later, hearts were harvested and neutrophils were detected with flow cytometry (**A**), and quantified (**B**). Number of cells shown as mean; sham n = 5, MI = 6; **P < 0.01 (*unpaired t test*). Bone marrow neutrophils were subjected to migration assay with modified Boyden chamber plates to CMC conditioned media. Number of migrated cells shown as mean ± SEM; n = 5; *P < 0.05; ***P < 0.001; ****P < 0.0001 (*one-way ANOVA*) (**C**). Bone marrow neutrophils were cultured in serum depleted media for 24 h in the presence or absence of CMCs conditioned media. Apoptotic cells were detected with Annexin V/PI staining (**D**) and quantified with flow cytometry (**E**). Number of apoptotic cell shown as mean ± SEM; n = 5; **P < 0.01; ****P < 0.0001 (*multiple t test*).

Neutrophils infiltrating ischemic myocardium secrete proteolytic enzymes that degrade extracellular matrix and dead cells, which supports efferocytosis and removal of dead tissue by macrophages^14,19^. Next, we evaluated the effect of CMCs on neutrophil infiltration. CMCs (0.5 × 10^6^) were injected intramyocardially 2 d after MI. At 7 d after cell injection, hearts were harvested and subjected to enzymatic digestion. The myocardial cell suspensions were stained with monoclonal antibodies and quantified with flow cytometry. We found that compared with vehicle controls the CD11b^high^Ly6G^high^ neutrophil count in hearts injected with CMCs was increased (69.7 ± 8.7 vs 242 ± 41.3 cells per mg of tissue, *P* < 0.01). Further analysis showed that both subpopulations of pro-inflammatory N1 (CD206^low^) and pro-reparative N2 (CD206^high^) neutrophils were significantly increased in response to CMC injection (N1, 26.1 ± 4.8 vs 95.2 ± 17.7, *P* < 0.01; N2, 46.7 ± 5.2 vs 136.9 ± 21.9 cells per mg of tissue, *P* < 0.01) (Fig. 2A, B). Taken together, these data indicate that CMC injection recruits both N1 and N2 neutrophil populations.

### CMC secretome has chemotactic and anti-apoptotic effects on neutrophils in vitro

Neutrophils are short-lived cells. Once they extravasate into the myocardium, they die by apoptosis or NETosis within a few hours. Their life span can be extended when exposed to pro-inflammatory cytokines. We hypothesized that the number of neutrophils in the heart after CMC injection increases both because of recruitment and prolonged cell survival. To test this hypothesis, we collected conditioned media from CMCs and performed chemotaxis assay with modified Boyden chamber plates. We found that CMC-conditioned media has a strong dose-dependent chemoattractant activity for neutrophils (Fig. 2C). Next, we tested the effect of CMC conditioned media on neutrophil survival. We isolated bone marrow neutrophils and subjected them to serum starvation-induced apoptosis. We detected apoptosis in neutrophils with Annexin V and PI staining and quantified it with flow cytometry (Fig. 2D). Serum starvation induced robust neutrophil apoptosis (52.9 ± 0.7% Annexin V^pos^ cells), which was significantly reduced in the presence of CMC-conditioned media (32.3 ± 1.2% Annexin V^Pos^ cells, *P* < 0.001) (Fig. 2E). Further stratification for early and late apoptosis with PI staining indicated that both phases of apoptosis were significantly reduced in the presence of CMC-conditioned media (Fig. 2E). Together, these data suggest that the increased number of neutrophils after CMC injection may be due to both increased migration and survival induced by CMC paracrine factors.

### CMC injection recruits Ly6C^high^ monocytes and increases macrophages in infarcted hearts

**Figure 3:**
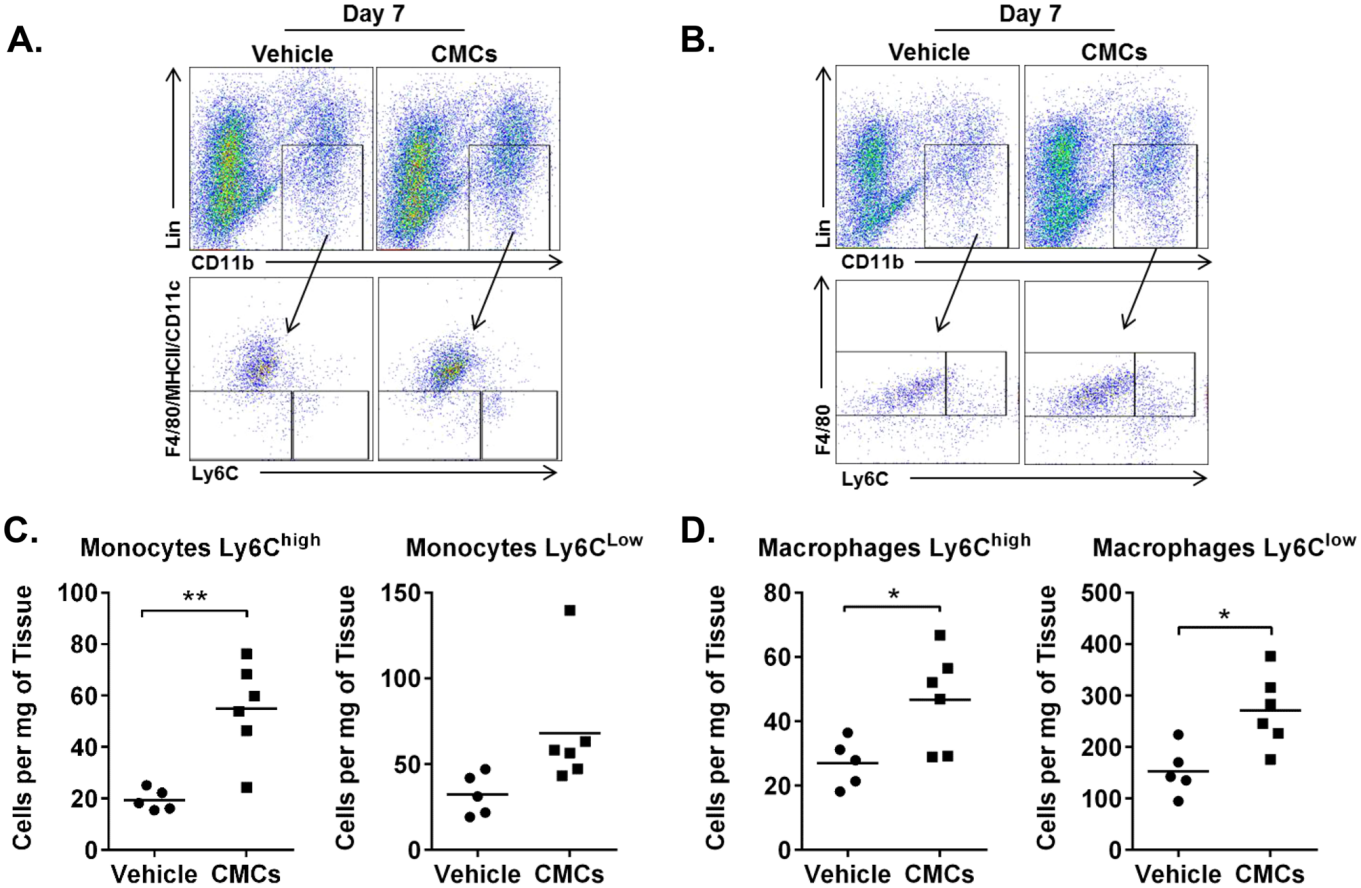
Intramyocardial injection of CMCs increases monocytes and macrophages in infarcted mouse hearts. CMCs (5 × 10^5^) were intramyocardially injected in the mouse hearts 2 d after MI. Seven days later, hearts were harvested and monocytes and macrophages were detected with flow cytometry (**A** and **B** respectively), and quantified (**C** and **D** respectively). Number of cells shown as mean; sham n = 5, MI n = 6; *P < 0.05; **P < 0.01 (*unpaired t test*).

Myocardial damage due to ischemia recruits two populations of monocytes, identified on the basis of Ly6C expression. Ly6C^high^ monocytes are proinflammatory, whereas Ly6C^low^ monocytes are patrolling monocytes with anti-inflammatory and pro-reparative cytokine profiles. Interestingly, the recruitment of both monocyte populations is essential for proper infarct healing and timely scar formation^18^. We found that intramyocardial administration of CMCs increased both Ly6C^high^ (vehicle 19.4 ± 1.8, CMCs 54.8 ± 7.5 cells per mg of tissue, *P* < 0.01) and Ly6C^low^ monocytes (vehicle 32.3 ± 5.5, CMCs 68.0 ± 14.61 cells per mg of tissue, *P* < 0.01) measured 7 days after cell injection (Fig. 3A, C). Next, we used flow cytometry to enumerate the number of macrophages in hearts injected with CMCs and vehicle. We stratified the macrophage population into two subpopulations based on the expression of Ly6C. We found that both Ly6C^high^ and Ly6C^low^ macrophages were significantly increased in CMC-injected hearts (Ly6C^high^ 27.0 ± 3.3 vs 46.7 ± 6.2, *P* < 0.05; Ly6C^low^ 153.2 ± 21.4 vs 270.4 ± 28.8 cells per mg of tissue, *P* < 0.05) (Fig. 3B, D). Because the total number of macrophages in post-MI hearts is determined by the infiltration of monocytes that further differentiate to macrophages and by the proliferation of already differentiated macrophages, we speculate that the increased number of macrophages in CMC-injected hearts is partially due to recruitment of monocytes.

### CMCs have a pro-migratory effect on bone marrow-derived monocytes

**Figure 4:**
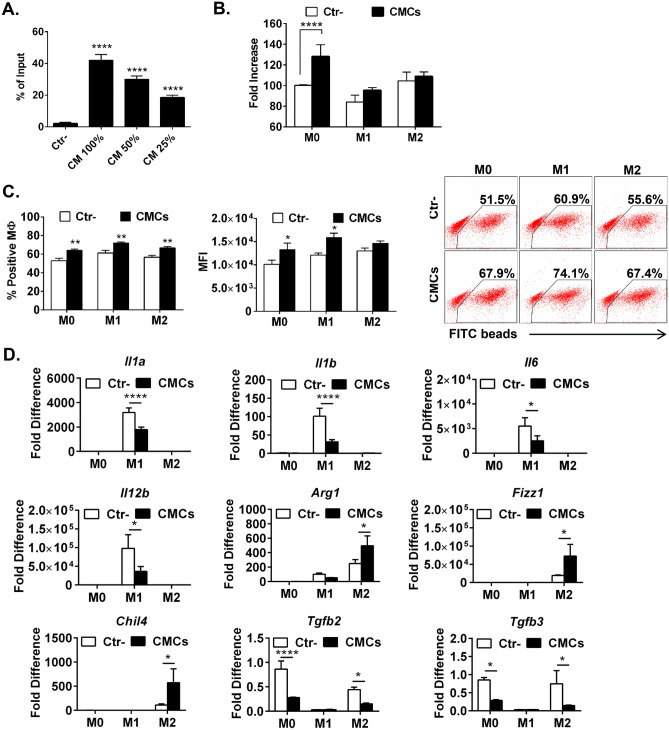
CMC secretome increases monocyte migration and macrophage proliferation, phagocytosis, and inhibits pro-inflammatory activation in vitro. Bone marrow monocytes were subjected to migration assay with modified Boyden chamber plates to CMC conditioned media. Number of migrated cells shown as mean ± SEM; n = 4; ****P < 0.0001 (*one-way ANOVA*) (**A**)**.** Naïve bone marrow macrophages (M0) were stimulated with LPS (100 ng/mL) and IFNγ (20 ng/mL) (M1), or IL-4 (10 ng/mL) and IL-13 (10 ng/mL) in the precence CMC conditioned media or control media. After 24 h macrophage number was evaluated with microscopy (**B**); phagocytosis of FITC IgG opsonized latex beads was determined with flow cytometry (**C**), and expression of pro-inflammatory gene expression was quantified with qPCR (**D**). Data are means ± SEM; n = 6; *P < 0.05; **P < 0.01; ****P < 0.0001 (*one-way ANOVA*).

Because we found in our in vivo experiments that CMC injection recruits monocytes, we reasoned that CMCs may induce monocyte migration via secretion of chemotactic factors. To test this hypothesis, we collected conditioned media from CMCs and used modified Boyden chamber plates to test the migratory activity of bone marrow monocytes. We found that monocytes exhibit a dose-dependent increase in migratory response to conditioned media (Fig. 4A). These data suggest that the secretome of CMCs can directly recruit immune cells, including monocytes and neutrophils, via secreted paracrine factors.

### The secretome of CMCs increases macrophage survival and phagocytosis, and reduces macrophage pro-inflammatory activation

The number of macrophages in the infarcted myocardium depends on the recruitment of monocytes, but also is regulated by proliferation and survival of cardiac monocyte-derived macrophages. As described above, we found that monocytes have a strong chemotactic response to CMC conditioned media. Here, we tested the effect of CMC secretome on the survival/proliferation of monocyte-derived macrophages in vitro. Bone marrow macrophages and CMCs were co-cultured using a transwell system in which secreted factors can be exchanged via a porous membrane without direct contact of co-cultured cells. We found that after a 24-h co-culture, the number of naïve macrophages was significantly increased in the presence of CMCs (~ 128% of control, *P* < 0.05). However, CMC secretome had no effect on M1 or M2 polarized macrophage survival/proliferation (Fig. 4B). The main role of macrophages as phagocytes in wound healing is to remove dead cells via phagocytosis. Next, we used the same co-culture system to evaluate macrophage phagocytosis. After a 24-h co-culture, transwells containing CMCs were removed and macrophages were stimulated with latex beads opsonized with FITC labeled IgG for 1 h. Subsequently, macrophages were detached from dishes and analyzed for the efficiency of phagocytosis with flow cytometry. We found that macrophages co-cultured with CMCs had increased phagocytic activity regardless of macrophage polarization status (M0, M1, or M2) (Fig. 4C).

Macrophages recruited to the damaged myocardium not only remove dead cells via phagocytosis, but also secrete cytokines that shape the immune response and regulate activation of fibroblasts and angiogenesis. In response to danger-associated molecular patterns (DAMPs), macrophages become activated and secrete pro-inflammatory cytokines, perpetuating inflammation and contributing to worsening cardiac function. To mimic the pro-inflammatory response of macrophage to DAMPs, we stimulated macrophages with LPS and IFN-γ, which elicited strong pro-inflammatory *Il1a*, *Il1b*, *Il6*, *and* *Il12b* gene expression in macrophage. In the presence of CMCs, the pro-inflammatory activation of CMCs was significantly reduced (Fig. 4D). Moreover, CMC conditioned media enhacend pro-reparative (*Arg1*, *Fizz1*, *Chil4*) and reduced pro-fibrotic (*Tgfb2* *and* *Tgfb3)* gene program induced with IL-4 and IL-13 (Fig. 4D). To determine the impact of CMCs on cardiac macrophages in vivo, we sorted macrophages from infarcted hearts 7 d after CMC or vehicle injection and subjected them to gene expression profiling with qPCR arrays. We found that macrophages isolated from hearts injected with CMCs exhibited numerous changes in gene expression related to immunoregulation and repair compared with vehicle-injected hearts (Supplementary Fig. 3). Taken together, these data indicate that CMCs have strong immunomodulatory effects on macrophages, both in vivo and in vitro. Administration of CMCs to infarcted hearts can regulate not only the number of macrophages, but also their function related to immunomodulation and tissue repair.

### CMCs have no effects on lymphocyte numbers in vivo

**Figure 5:**
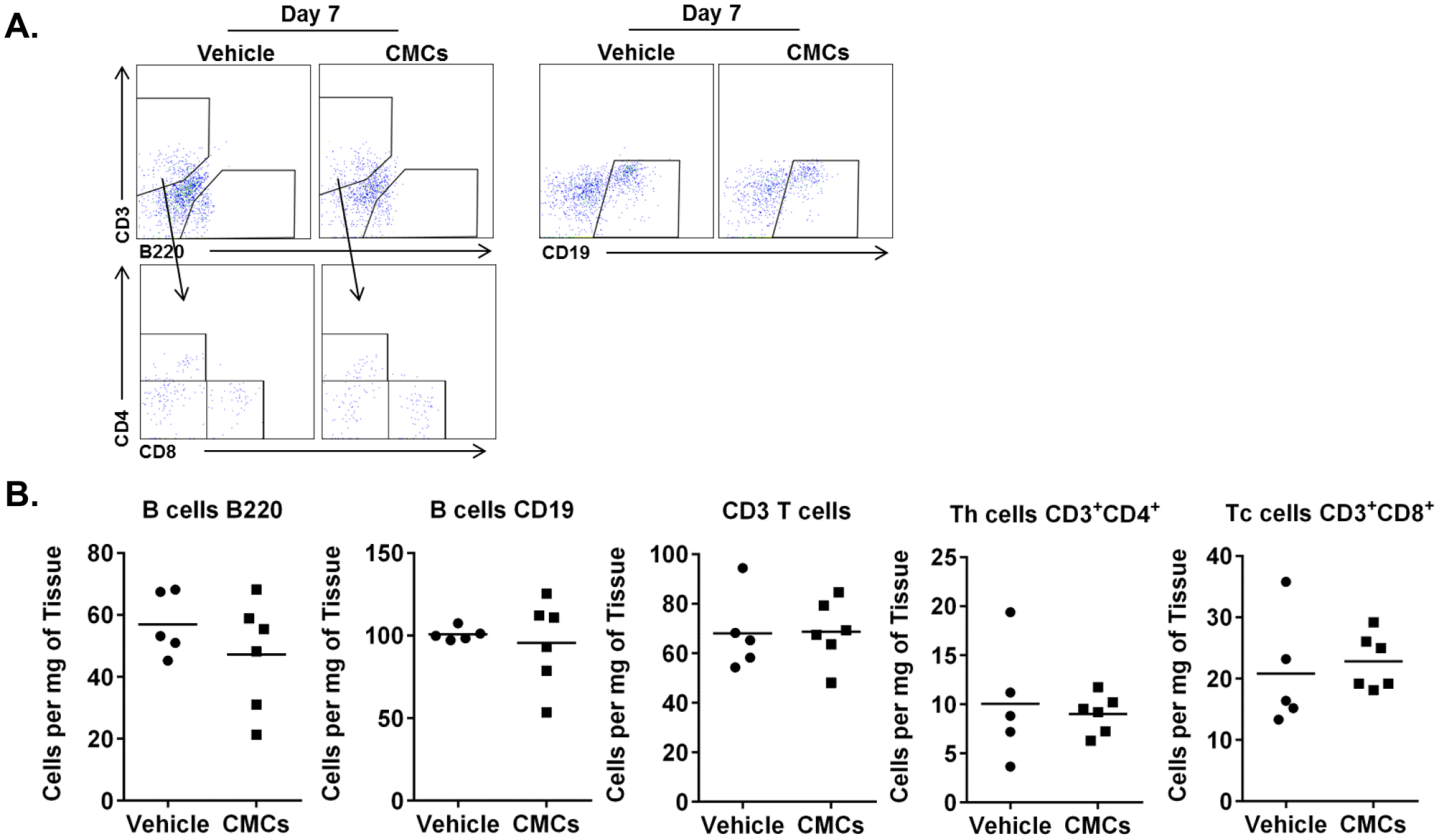
Intramyocardial injection of CMCs has no effect on B and T cell number in vivo. CMCs (5 × 10^5^) were intramyocardially injected in the mouse hearts 2 d after MI. Seven days later, hearts were harvested and B and T cells were detected with flow cytometry (**A**), and quantified (**B**). Number of cells shown as mean; sham n = 5, MI n = 6; (*unpaired t test*).

Both B and T lymphocytes are involved in infarct healing and scar formation^14^. Here, we tested the impact of CMC injection on B and T cell infiltration into infarcted mouse hearts. We found that B lymphocytes expressing CD19 or B220 did not increase in the myocardium after CMC injection. Similarly, the total number of T lymphocytes (CD3^pos^) was not changed after CMC injection, and there was no effect on Tc CD8^pos^ or Th CD4^pos^ lymphocyte recruitment (Fig. 5). These data indicate that, after intramyocardial injection, CMCs secrete factors that recruit innate immune cells but not B or T lymphocytes.

### CMCs express CC and CXC chemokines regulating migration of neutrophils and monocytes

**Figure. 6:**
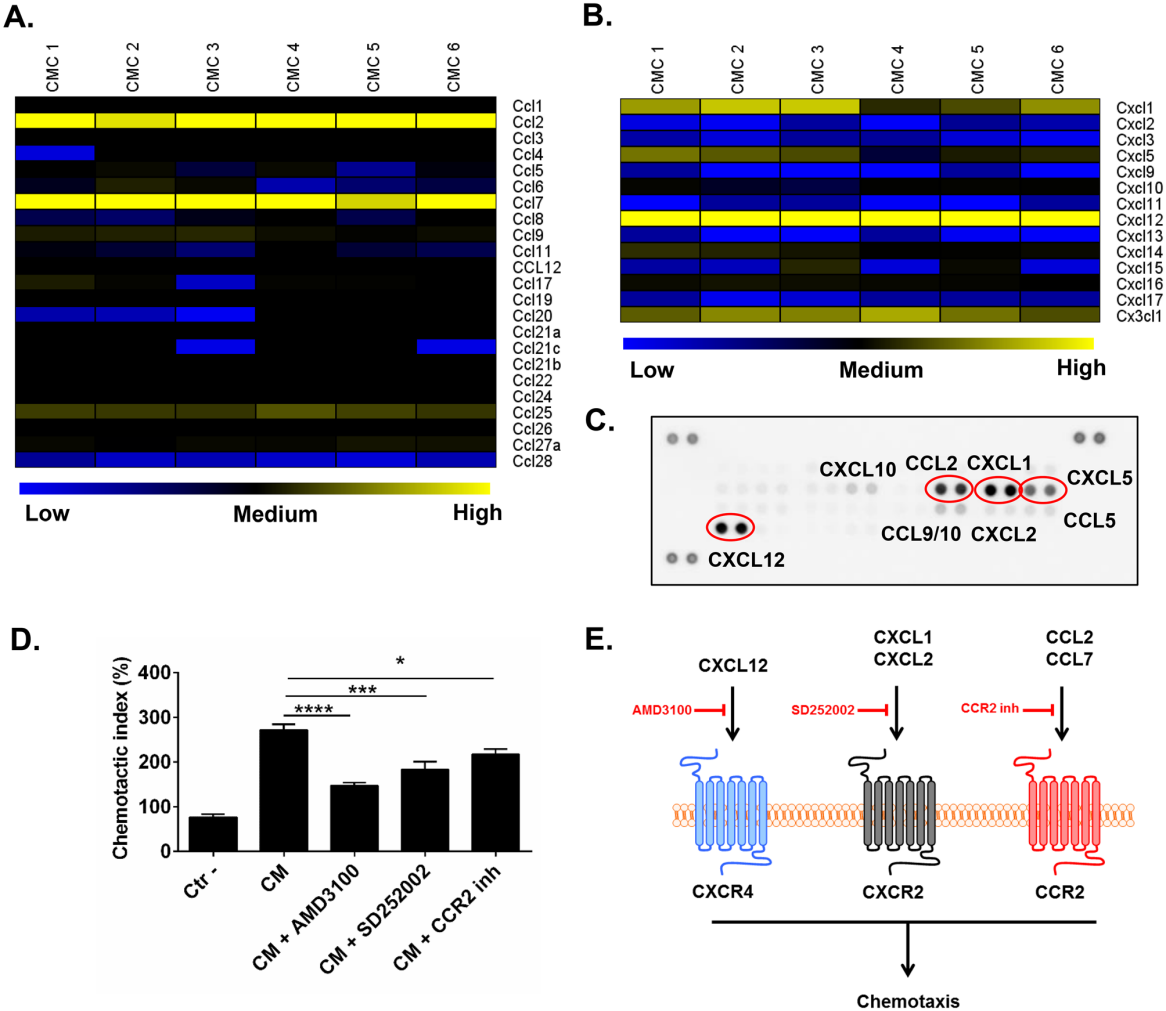
CMCs secrete chemokines for neutrophils and monocytes. Expression of CC (**A**) and CXC chemokines (**B**) was determined with RNAseq. Expression of CC and CXC chemokines in CMC conditioned media was evaluated with Chemokine Array (**C**). Bone marrow cells were subjected to migration assay with modified Boyden chamber plates to CMC conditioned media in the presence of AMD3100, SD252002, or CCR2 inhibitor (**D**). Number of migrated cells shown as mean ± SEM; n = 4; *P < 0.05; ***P < 0.001; ****P < 0.0001 (*one-way ANOVA*). Schematic of chemokine-chemokine receptor interaction (**E**).

Monocyte and neutrophil migration is regulated by numerous CC and CXC chemokines^13,20^. We performed RNAseq and mined the data to detect CC and CXC chemokines. We found that CCL2, CCL7, CCL25, CXCL1, and CXCL12 were highly expressed at the mRNA level in CMCs (Fig. 6A, B). Next, we validated expression of these chemokines in CMC-conditioned media with chemokine protein array. We found particulary strong signal for CXCL1, CXCL12, CCL2, and CCL5 chemokines; and positive but weak signal for CXCL2, CXCL10, CCL5, and CCL9/10 (Fig. 6C). All these chemokines have been implicated in regulation of immune cell trafficking via activation of specific receptors. Next, we used chemokine receptor antagonists to identify which of the CMC-sectered chemokine contributes to chemotaxis of bone marrow immune cells. We found significant reduction of chemotactic activity of bone marrow cells to CMC-conditioned media after inhibition of CXCR4 (AMD3100), CXCR2 (SD 252,002), and CCR2 (CCR2 inhibitor) with specific small molecule receptor antagonists (Fig. 6D). These data suggest that the CXCL12-CXCR4, CXCL1/CXCL2-CXCR2, and CCL2/CCL7-CCR2 chemokine-chemokine receptor axes are involved in migration of neutrophils and monocytes to the infarcted heart after CMC administration (Fig. 6E). This hypothesis would have to tested in further experiments in vivo.

## Discussion

Our recent studies^4,5,26^ have shown that administration of CMCs improves cardiac function after MI without long-term engraftment or regeneration. Because the immune system plays an importand role in myocardial healing, the main goal of this work was to test the immunomodulatory properties of CMCs in vitro and in vivo. The main results can be summarized as follows: (1) at the time of CMC injection (2 d after a reperfused MI), the infarcted murine myocardium is infiltrated by neutrophils, monocytes, macrophages with pro-inflammatory phenotype, and cytotoxic T cells; (2) expression of genes for both pro- and anti-inflammatory cytokines is significantly increased at this time; (3) Intramyocardial CMC injection further increases recruitment of neutrophils, monocytes, and macrophages, whereas the number of B and T lymphocytes remains unchanged; (4) in vitro, the CMC secretome exerts strong chemotactic and pro-survival actions on neutrophils, induces migration of monocytes, increases survival and phagocytosis of macrophages, decreases pro-inflammatory activation of macrophages in response to LPS and IFNγ, and enhances a pro-reparative gene program after IL-4 and IL-13 stimulation. Taken together, these data demonstrate that CMCs have strong, heretofore unknown immunomodulatory properties for myeloid cells both in vitro and in vivo, but have no effects on B and T cells.

Although previous investigations have suggested that cell therapy affects innate immunity after acute MI^1,35^, to our knowledge this is the first study to identify immunoregulatory actions of CMCs in this setting. The data reveal a potential mechanism that may underlie the beneficial actions of CMCs in acute MI^5^. The study by Vagnozzi et al^35^ showed that intramyocardial injection of bone marrow MNC and c-kit^pos^ cardiac progenitors recruits monocytes and macrophages in naïve hearts. In our studies, we profiled immune cell infiltration in response to CMC injection in infarcted hearts. This was also the first study to indentify changes in a broad spectrum of immune cells (neutrophils, monocytes, macrophages, B and T cells) in response to CMCs in the setting of acute MI.

The immune response after acute MI is a dynamic and finely regulated series of events that culminates in scar formation. Tissue necrosis caused by myocardial ischemia elicits a strong immune response via activation of the complement system, production of reactive oxygen species (ROS), release of DAMPs, and secretion of chemokines^13–15,17,20^. In mouse hearts, this phase begins within minutes and lasts for up to 4 days after a non-reperfused infarction^13,14,17–20^. In our study, we used ischemia followed by reperfusion, which is known to result in faster resolution than permanent coronary ligation^17^. The reperfused MI model is more relevant to the clinical situation because in contemporary medical practice almost all MIs are reperfused, either spontaneously or iatrogenically. We found that 2 d after reperfusion and at the time of cell injection, the myocardium was still heavily infiltrated by pro-inflammatory N1 neutrophils and Ly6C^high^ monocytes, suggesting that CMC injection occurred during the initial pro-inflammatory phase. This conclusion is further supported by the expression of genes encoding the pro-inflammatory cytokines *Il1b*, *Il6*, *Tnfa* and the chemokine *Ccl2*. This initial pro-inflammatory period resolves within a few days and is followed by a reparative phase dominated by macrophages, lymphocytes, and proliferating scar-forming myofibroblasts^13,14,20–22,30^. The absence of B and T lymphocytes at 2 d after reperfusion further supports the conclusion that CMC injection occurred during the initial pro-inflammatory phase. Because at the time of CMC injection the myocardium is infiltrated with neutrophils, monocytes, and macrophages, but not with B and T cells, we decided to focus mainly on the interaction between CMCs with myeloid immune cells.

Neutrophils are the first immune cells infiltrating myocardium after infarction. Recruited neutrophils secrete proteolitic enzymes (hydrolases, elastase, neutral serine proteases, lysozyme, cathepsins), which degrade necrotic tissue and facilitate clearance of dead cells and extracellular matrix debris. Moreover, extravasated neutrophils produce proinflammatory mediators including cytokines (IL-1, IL-6, TNF-α), ROS, and extracellular traps that perpetuate the inflammatory response, thereby producing cytoxic effects and further contributing to myocardial injury^13,14,36,37^. Neverthless, neutrophil recruitment is necessary for proper myocardial recovery after infarction. Systemic neutrophil depletion leads to dysregulated monocytes/macrophage function, promotes excess ECM deposition, and worsens cardiac function^19^. Therefore, neutrophils can have a dual role in the process of myocardial healing.

Little is known about the effect of cell therapy on neutrophil function after MI. We found that intramyocardial injection of CMCs increased the number of both N1 and N2 neutrophil subpopulations, and that the CMC secretome exerts strong chemotactic and pro-survival activity on neutrophils. This suggests that the increased number of neutrophils after CMC injection could be caused by the CMC secretome though both mechanisms. Previous studies have shown that bone marrow MSCs exert immunomodulatory actions on neutrophils similar to CMCs. For example, in vitro experiments have demonstrated that co-culture of bone marrow MSCs increases survival of neutrophils; this anti-apoptotic effect of MSCs was mediated by secretion of IL-6, IFN-β, and GM-CSF^38^. In contrast, intramyocardial injection of human cord blood mononuclear cells, which contain both hematopietic and mesenchymal cells, in immunocompetent rats at the time of permanent coronary ligation leads to reduced infiltration of neutrophils^39^. This apparent discrepancy with our study could be due to numerous differences,including cell type, model of MI, time of cell administration, and follow-up interval for evaluation of immune cell infiltration^39^. The mechanism of increased neutrophil migration after CMC injection is unclear. We found that CMCs secrete numberous chemokines that act on neutrophils, including CXCL12, CCL2, CXCL1, and CXCL2. Determining the role of these CMC-secreted chemokines in the recruitment of neutrophils will require further in vivo and in vitro investigations.

Recent studies have established a critical role of monocytes/macrophages in myocardial repair after infarction^14,15,18,23–25^. Monocytes recruitment to the infarcted myocardium is a dynamic process which occurs in two phases. In the first phase, recruitment of pro-inflammatory Ly6C^high^ monocytes promotes tissue degradation though secreted proteases. Reparative Ly6C^low^ monocytes are then recruited to resolve inflammation and promote infarct healing^14,18^. As the pro-inflammatory phase resolves, Ly6C^high^ monocytes differentiate to reparative Ly6C^low^ macrophages, which promote wound repair and scar formation. We found that intramyocardial injection of CMCs facilitates recruitment of both Ly6C^high^ and Ly6C^low^ monocytes. That this process is mediated by chemokines produced by CMCs is supported by our finding that monocytes have strong chemotactic activity to CMC-conditioned media. Moreover, we found that CMCs secrete CCL2 and CX3CL1, which are strong chemoattractans for Ly6C^high^ and Ly6C^low^ monocytes, respectively^14,18,40^. Further studies will be necessary to determine the role of CMC-secreted chemokines in the recruitment of monocytes in vivo.

The number of macrophages in the heart after MI is regulated both by recruitment of monocytes and proliferation of local cardiac macrophages^41^. Our finding that CMC injection increases the myocardial content of Ly6C^low^ macrophages could be explained by increased recruitment of monocytes (discusses above) or enhanced proliferation/survival of cardiac macrophages. Since our in vitro studies show that co-culture with CMCs increases macrophage survival/proliferation, this could be a contributing mechanism to the increased number of macrophages. The macrophages recruited after MI can orchestrate the immune response by secreting proinflammatory cytokines (IL-1, IL-6, TNF-α) and chemokines (CCL2); in addition, they can promote repair and scar formation via secretion of cytokines that activate myofibroblast differentation (TGFβ and IL-10), via angiogenesis (VEGF), and via release of MMPs and TIMPs that regulate the extracellular matrix (ECM) network^15,23,40^. The macrophage shift from inflammatory to reparative phenotypes may be a direct consequence of macrophage function: efferocytosis, the uptake of apoptotic cells, reduced pro-inflammatory cytokine production and upregulated pro-reparative and anti-inflammatory cytokines (IL-10 and TGF-β)^42^. Our finding that macrophages co-cultured with CMCs exhibit increased phagocytosis suggests that CMC-secreted factors facilitate apoptotic cell removal and reduce pro-inflammatory cytokine production. In separate experiments, we found that co-culture with CMCs inhibits the pro-inflammatory activation of macrophages in response to LPS and IFN-γ. This suggests that CMCs enhance recruitment of macrophages, but also facilitate their phenotype swich directly by modulating their response to pro-inflammatory stimuli and also indirectly by facilitating their phagocytosis.

Similar immunomodulatory effects have been reported using other cells types. In in vitro co-culture systems, it has been shown that the secretome of BM MSCs facilitates switch of macrophages from pro-inflammatory to pro-reparative and enhaces their phagocytic activity^43^. Several MSC-derived factors have been identified that modulate macrophage function, including prostaglandin E2, indoleamine 2,3-dioxygenase, TGF-β, and IL-10^1,44–46^. Furthermore, infusion of BM MSCs at 2 d after MI in mice induces a macrophage switch from pro-inflammatory toward a reparative phenotype^43^. In addition, the secretome of cardiosphere-derived cells (CDCs) has been shown to activate the reparative program in macrophages and decrease the expression of CD80 and CD86, which are co-stimulatory receptors necessary for T cell activation. In vivo injection of CDCs in rats soon after reperfuction results in improvement of LV function, but also decreases the number of myocardial CD68^pos^ macrophages^47,48^. These studies further support the conclusion that cell therapy has immunomodulatory effects. It seems likely that the time of cell injection has an impact on immune cell recruitment.

Our data and reports by others^1,38,39,43,46–48^ indicate that cell therapy has immunomodulatory actions on macrophages and neutrophils, both in vivo and in vitro. However, whether these actions are important for the beneficial effects of cell therapy remains unclear. Since neutrophils and macrophages play a key role in wound repair and scar formation after MI, and since systemic depletion of neutrophils or macrophages causes dysregulated immune response, impaired scar formation, and decreased cardiac function after MI^16,18,19,23,25^, immune cell depletion would not be an optimal strategy to address the question of whether the salutary effects of cell therapy rely on immunomodulatory actions on neutrophils and macrophages. Such a strategy, however, may be useful in models of chronic heart failure, in which cells are infused after the acute inflammatory response has resolved and temporary depletion of neutrophils or macrophages would not have a substantial impact on cardiac function. Another challenge is to identify the immunomodulatory mediators that are secreted by the injected cells. Since CMCs secrete numerous factors that can work synergistically to modulate the immune system, it may be difficult to pinpoint the factor(s) responsible for the immunomodulatory and salutary effects of the injected cells.

The present results provide new insights into the actions of CMCs transplanted in the acute phase of MI. The modulatory effects of CMCs on innate, but not adaptive, immune cells described herein could facilitate elucidation of the mechanism of action of CMC-based therapy and cell therapy in general. Ultimately, improved understanding of the immunomodulatory actions of cell therapy may help harness the immune system to ameliorate the outcome of ischemic heart disease.

## Supplementary information

Supplementary information.
