# Supplementary material for: Administration of cardiac mesenchymal cells modulates innate immunity in the acute phase of myocardial infarction in mice
Source: Sci Rep. 2020 Sep 8;10:14754. doi: 10.1038/s41598-020-71580-z (PMC7479609; doi:10.1038/s41598-020-71580-z)
Supplement: Supplementary file 1 — Supplementary information. [file 41598_2020_71580_MOESM1_ESM.pptx]

## Slide 1
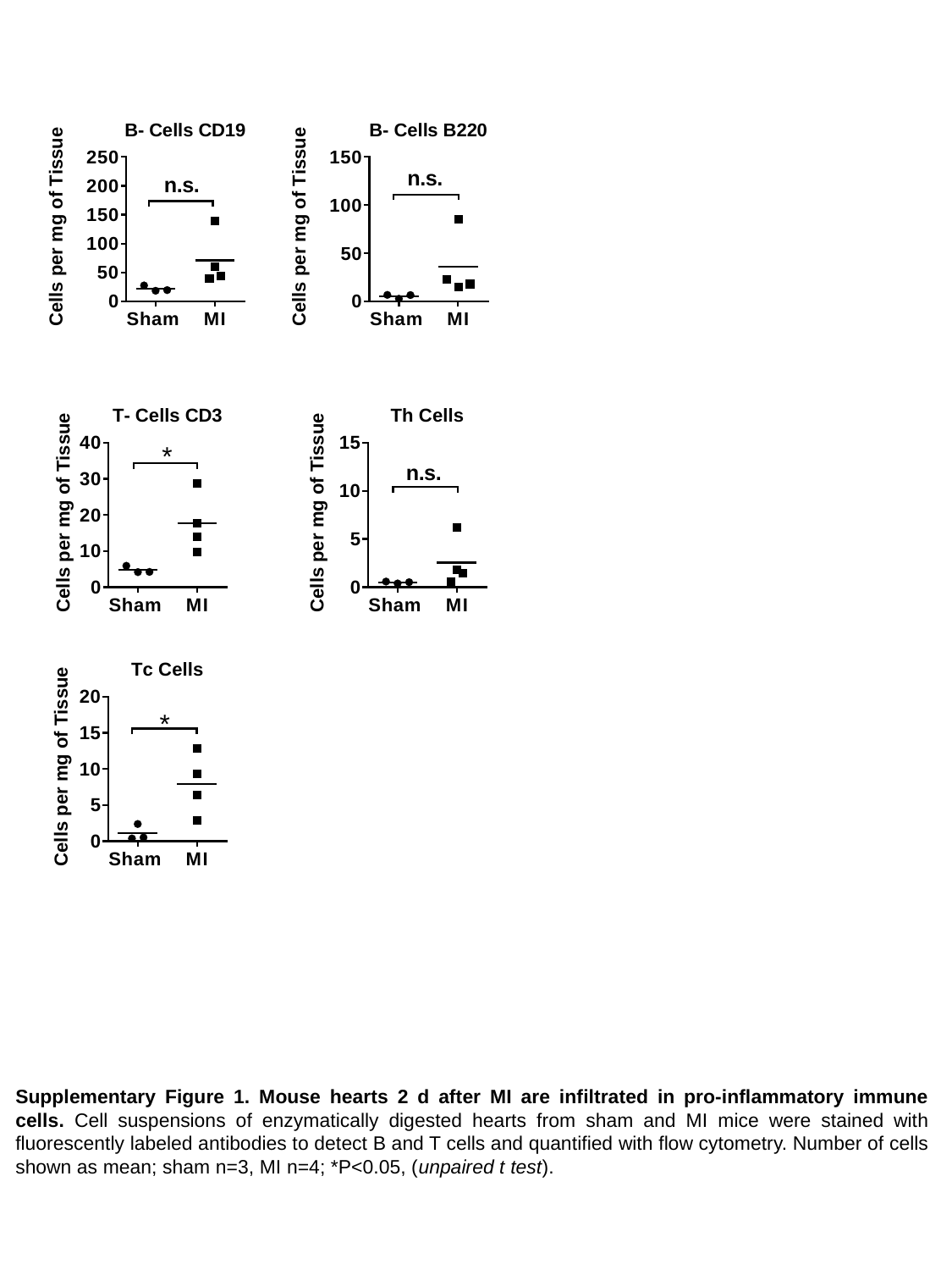

Supplementary Figure 1. Mouse hearts 2 d after MI are infiltrated in pro-inflammatory immune cells. Cell suspensions of enzymatically digested hearts from sham and MI mice were stained with fluorescently labeled antibodies to detect B and T cells and quantified with flow cytometry. Number of cells shown as mean; sham n=3, MI n=4; *P<0.05, (unpaired t test).

## Slide 2
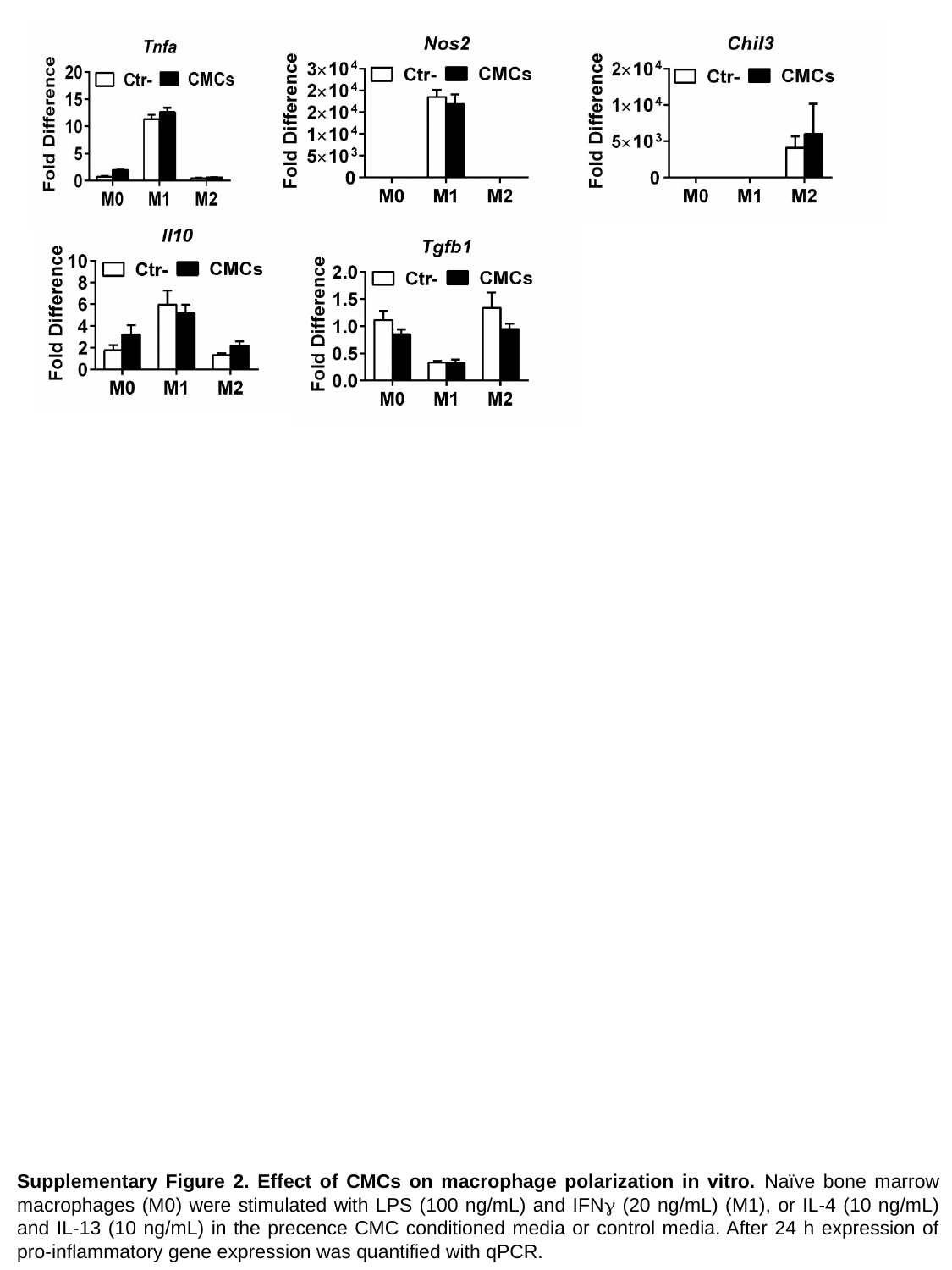

Supplementary Figure 2. Effect of CMCs on macrophage polarization in vitro. Naïve bone marrow macrophages (M0) were stimulated with LPS (100 ng/mL) and IFNγ (20 ng/mL) (M1), or IL-4 (10 ng/mL) and IL-13 (10 ng/mL) in the precence CMC conditioned media or control media. After 24 h expression of pro-inflammatory gene expression was quantified with qPCR.

## Slide 3
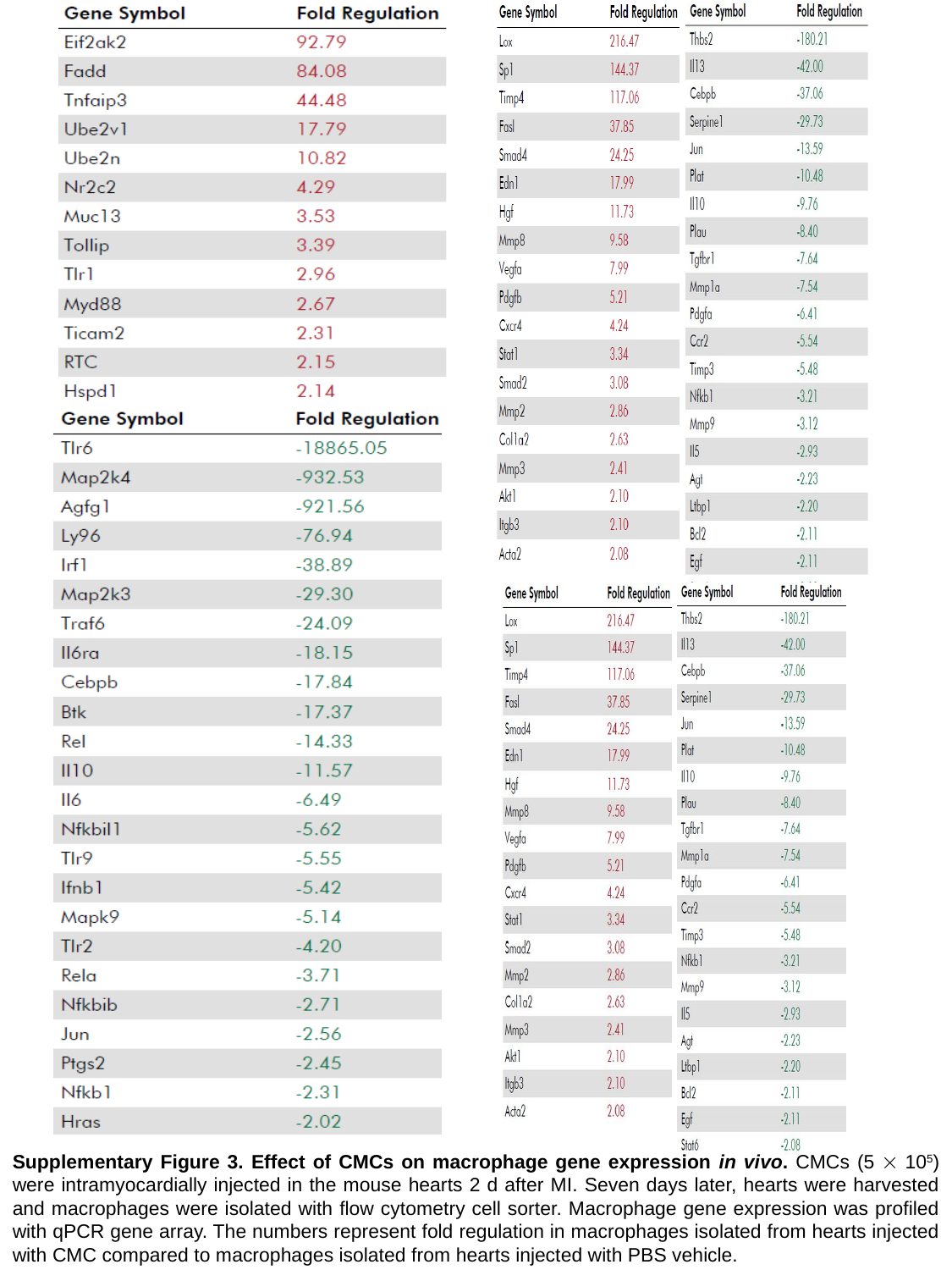

Supplementary Figure 3. Effect of CMCs on macrophage gene expression in vivo. CMCs (5 × 10^5^) were intramyocardially injected in the mouse hearts 2 d after MI. Seven days later, hearts were harvested and macrophages were isolated with flow cytometry cell sorter. Macrophage gene expression was profiled with qPCR gene array. The numbers represent fold regulation in macrophages isolated from hearts injected with CMC compared to macrophages isolated from hearts injected with PBS vehicle.
